# Effect of the enzyme and PCR conditions on the quality of high-throughput DNA sequencing results

**DOI:** 10.1038/srep08056

**Published:** 2015-01-27

**Authors:** Claudia Brandariz-Fontes, Miguel Camacho-Sanchez, Carles Vilà, José Luis Vega-Pla, Ciro Rico, Jennifer A. Leonard

**Affiliations:** 1 Conservation and Evolutionary Genetics Group, Estación Biológica de Doñana (EBD-CSIC), Sevilla, Spain; 2 Facultad de Medicina Veterinaria, Universidad de Panamá, Panama; 3 Laboratorio de Investigación Aplicada, Cría Caballar de las Fuerzas Armadas, Córdoba, Spain; 4 University of the South Pacific, School of Marine Studies, Faculty of Science, Technology & Environment, Laucala Campus, Suva, Fiji

## Abstract

Library preparation protocols for high-throughput DNA sequencing (HTS) include amplification steps in which errors can build up. In order to have confidence in the sequencing data, it is important to understand the effects of different *Taq* polymerases and PCR amplification protocols on the DNA molecules sequenced. We compared thirteen enzymes in three different marker systems: simple, single copy nuclear gene and complex multi-gene family. We also tested a modified PCR protocol, which has been suggested to reduce errors associated with amplification steps. We find that enzyme choice has a large impact on the proportion of correct sequences recovered. The most complex marker systems yielded fewer correct reads, and the proportion of correct reads was greatly affected by the enzyme used. Modified cycling conditions did reduce the number of incorrect sequences obtained in some cases, but enzyme had a much greater impact on the number of correct reads. Thus, the coverage required for the safe identification of genotypes using one of the low quality enzymes could be seven times larger than with more efficient enzymes in a biallelic system with equal amplification of the two alleles. Consequently, enzyme selection for downstream HTS has important consequences, especially in complex genetic systems.

High throughput DNA sequencing (HTS) has dramatically reduced the cost per base sequenced^1^. HTS technologies, however, are fundamentally different from Sanger sequencing and face different problems. In HTS single molecules of DNA yield sequences, as opposed to a large pool of molecules in Sanger sequencing. This exposes errors that can occur during library preparation. For example, errors could result from the misincorporation of nucleotides during the amplification steps of library preparation. During amplification there can be partial synthesis of a DNA strand that can act as a primer in a downstream polymerase chain reaction (PCR) cycle and form a chimeric sequence if it amplifies a related allele. These sources of errors originating in PCR amplification are poorly characterized, but increasingly recognized as a problem^2^,^3^.

Recent technical advances in HTS yielding longer reads of 350 to 1000 base pairs (bp) and methodological advances such as the incorporation of index sequences allow multiple targeted loci from many individuals to be sequenced simultaneously^4^,^5^,^6^. Targeted loci could have different characteristics. The simplest systems, such as loci in the mitochondrial DNA, Y chromosome (in mammals) or W chromosome (in birds) loci, are expected to yield a single haplotype and are thus the easiest to determine the sequence of. Most single copy nuclear markers, which are potentially biallelic in diploid organisms, are more challenging to accurately genotype. Very complex systems, such as gene families in which many different alleles could be present in a single individual, can be very difficult to accurately characterize. PCR based errors have been shown to be a problem in the characterization of polygenic immune system loci in model organisms^2^,^3^. Accurately genotyping complex loci in non-model systems for which there is not a lot of comparative data to verify results can be even more challenging^7^,^8^,^9^,^10^.

One factor that could play an important role in identifying correct alleles and genotypes using HTS approaches is the enzyme used in the DNA amplification. In this study we tested the ability of thirteen different enzymes to yield the true sequence(s) via HTS in three genetic marker systems of different complexity. We also tested if modified PCR conditions could increase the yield of correct templates, as suggested in previous studies^11^,^12^,^13^,^14^,^15^. Understanding the frequency and potential sources of erroneous sequences is of prime importance for the design of optimal protocols in HTS approaches to characterize genetic diversity in individuals and populations, and is even more critical in non-model systems.

## Results

We tested the ability of 13 different enzymes to yield the true sequence(s) in three different marker sets of varying complexity (see Methods, Table 1 for abbreviations). The three sets we used were: Test 1, mitochondrial DNA from wolves, expected to yield a single sequence per individual; Test 2, MHC class II exon 2 (MHC II) in horses, a single copy nuclear gene with one or two alleles per individual; and Test 3, MHC class I exon 3 (MHC I) in horses, a multigene family which could yield several alleles per individual. Three different individuals were included in each test. A further two tests (Tests 2b and 3b) were designed to evaluate the ability of modified PCR cycling conditions to reduce amplification-associated errors. These tests were done only with the two more complex systems: MHC II for Test 2b and MHC I for Test 3b.

Error patterns and rates can vary between sequencing platforms^1^,^16^, and even independent runs in the same platform can have an effect on the genotypes^17^. Here we focus on the performance of different polymerase on a single platform in order to more reliably assess to what degree this is an important factor to take into account when designing experiments. We chose the Roche 454 Junior sequencing platform. This platform is appropriate for this experiment because it allows relatively long and variable read lengths, so the entire length of the three different PCR products could be sequenced simultaneously in single reads.

Six enzymes (Phusion, Gold, FastStart, Roche Taq, HotStar and Biotaq) worked across all tests, five of them (Velocity, OneTaq, Imax, KapHF and Pwo) worked inconsistently in different tests. We were not able to get Vent or DeepVent to amplify in any of the systems after 12–29 tries each for Tests 1–3. The sequencing run produced 102,484 reads, from which 63,942 passed size (full length) and quality filters (complete MID and primer sequences) and could be successfully assigned to the experimental units (Genetic system/Enzyme/PCR condition/Biological replica) yielding an average coverage of 566, although with a large variation (standard deviation, s. d. = 1900). The average coverage for the sequences used in Test 1 was 1004 (s. d. = 3225), 203 in Test 2 (s. d. = 194), 370 in Test 3 (s. d. = 484), 834 in Test 2b (s. d. = 2300) and 337 in Test 3b (s. d. = 546). Eleven of the 13 enzymes tested yielded a band of the expected size in Test 1, eight in Test 2, eight in Test 3, six in Test 2b and six in Test 3b.

**Figure 1:**
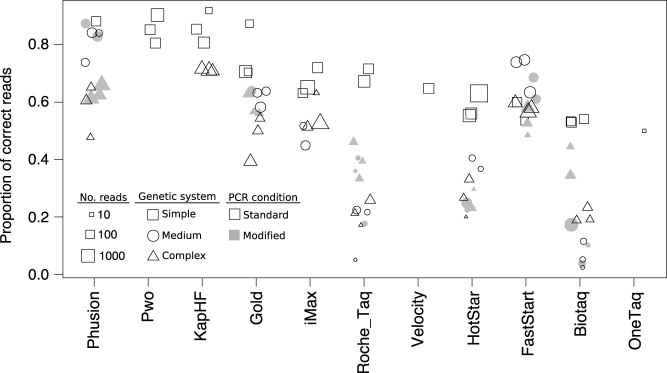
Proportion of correct reads for the three genetic systems (simple: a single allele per individual, *squares*; medium: two alleles, *circles*; and complex: multiple alleles, *triangles*) using standard PCR conditions (*open*) and modified PCR conditions to reduce chimera formation (*gray*). The size of the shape is indicative of the number of reads (see legend). All enzymes yielded at least 50% correct reads in the simplest system, mitochondrial DNA (Test 1; open squares). Some enzymes only worked for a given set of conditions (cycling conditions/genetic system). A group of enzymes consisting of Phusion, Gold and FastStart yielded a high proportion of correct reads cosistently accross all conditions. Others, such as Roche Taq, HotStar and Biotaq, yielded a low percent of correct reads for the more complex systems (MHC class I and MHC class II). Abbreviations as defined in Table 1.

There was a significant effect of the enzyme on the quality of the sequences obtained (proportion of reads with a correct sequence) for all tests (p < 0.001 in all cases). In general, Biotaq produced the lowest portion of correct reads across all tests whereas Phusion, Pwo and KapHF worked best (Supplementary File 1). For Test 1 (with only one allele expected per individual), all the enzymes that successfully amplified DNA (11 out of 13) yielded from 50–53% (OneTaq and Biotaq) to 88–92% (Phusion, Pwo and KapHF) correct reads (Figure 1). For Test 2, the proportion of correct reads was on average 23% lower than for Test 1. There was also more variation between the enzymes, with correct reads ranging from 2% (Biotaq) to 84% (Phusion) (Figure 1). For Test 3, the multigene family marker system, the recovery of correct sequences ranged from 17–20% (Biotaq, HotStar and Roche *Taq*) to 65–71% (Phusion and FastStart) (Figure 1).

For the system with up to 2 alleles, the modified PCR had no effect on the proportion of correct reads (p = 0.31, Test 2 vs Test 2b). For the complex system, the multigene family, the proportion of correct reads was significantly higher under the modified PCR conditions, by an average of 7.5% (p < 0.001, Test 3 vs Test 3b).

**Figure 2:**
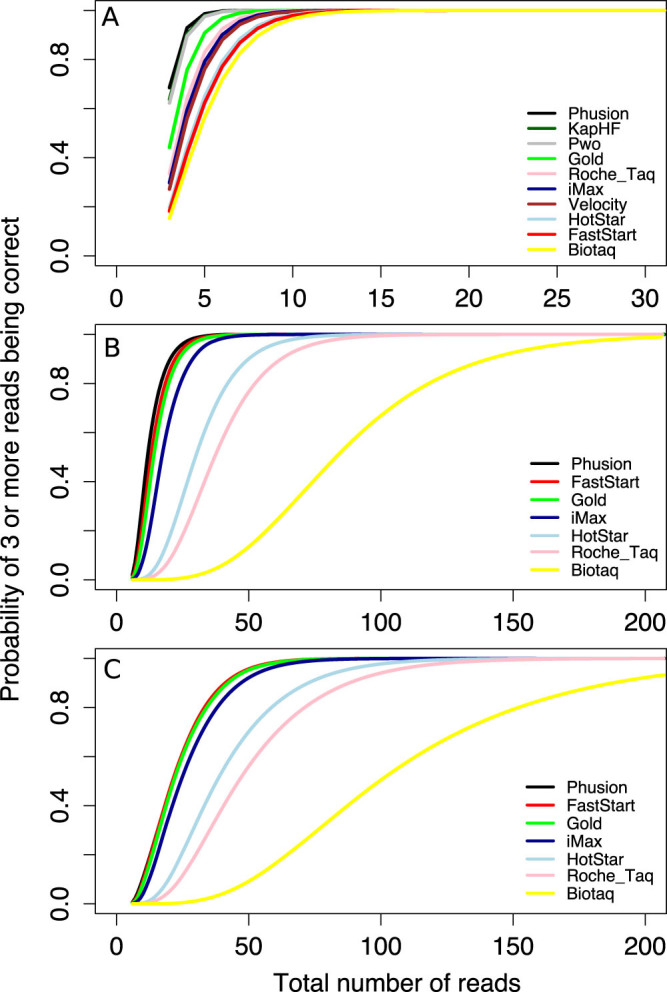
Probability of obtaining 3 or more correct sequences for a given number of reads based on the proportion of correct reads observed for each enzyme and genetic system. (A). For the simplest genetic system, with only one allele per individual. (B). For a locus with two alleles that amplify equally well (3 or more correct sequences for each of the two alleles). (C). For a locus with two alleles where one amplifies twice as well as the other. Note that the scale on the X-axis in panel A is different from that in B and C.

We used the proportion of correct sequences obtained with each enzyme from Tests 1, 2 and 2b to calculate the probability of obtaining three or more copies of the correct allele(s). We simulated this for a simple system, a haplotype (data from Test 1), and for a more complex system, a single locus with two alleles (combined data from Tests 2 & 2b). Unequal amplification of alleles in PCR reactions where more than one allele are amplified has been observed widely^18^,^19^. For this reason we also simulated the number of reads needed to reach the same level of confidence when one allele in the two allele system amplified twice as well as the other. For the haplotype, between 7 (for Phusion, Pwo and KapHF) and 16 (Biotaq) reads were enough to have a 99.9% probability of obtaining 3 or more correct sequences (Table 1, Figure 2A). However, the number of reads required increased sharply as the gene system got more complex. For two alleles that amplify equally, between 42 (for Phusion) and 271 (Biotaq) reads were needed to have 99.9% confidence of getting three correct copies for each of the two alleles (Table 1, Figure 2B). In the case of unequal amplification, the coverage necessary increased to 87 for Phusion, and to 395 for Biotaq (Table 1, Figure 2C).

## Discussion

The *Taq* polymerase enzyme used in the PCR steps of library preparation for HTS had a very important impact on the proportion of correct reads after sequencing. In the simplest case of a single allele being present, as in mitochondrial DNA or sex specific chromosome markers (Test 1), the majority of the reads (50–92%, depending on the enzyme) for all enzymes that worked (11 out of 13) had the correct sequence. In this marker system, high confidence that the haplotype identified is accurate was achieved with a low coverage of 7× for the best enzymes and 16× for the worst.

However, the proportion of correct reads went down in multi- allelic systems. In the just slightly more complex system of a single copy nuclear gene with two alleles (Test 2), the proportion of correct reads went down by an average of 23% (Figure 1). Calculations based on the proportion of sequences with the correct sequence revealed that for the best enzymes, and assuming equal amplification of the two alleles, 42 to 48 reads are necessary to have a high confidence in the identification of genotypes (probabilities of 99.9% or higher for the identification of each allele). The coverage required for the worse enzymes was much larger (above 270×).

This difference became even more pronounced when the model was more realistic and one allele amplified twice as well as the other. In this case, the coverage necessary to reach a similar degree of confidence in the results as for equal amplification of the alleles almost doubled. Differences in the amplification success of the two alleles in a biallelic system can realistically be much larger than a ratio of 1:2. Thus, the coverage necessary to have high confidence in a genotype would also be much higher. Cycling conditions also had a significant effect on the proportion of correct reads in some cases, but the effect was of a much smaller magnitude than the effect of the enzymes.

The results presented here suggest that for the best enzymes, under the most favorable PCR amplification conditions, and perfectly equal amplification of the two alleles of a single copy nuclear gene in a diploid organism, 42 to 48 reads are necessary to have a high confidence in the identification of genotypes. For other enzymes and in the more realistic case of unequal amplification of the alleles, nearly 400 reads are necessary to reach a similar degree of confidence in the results. This greatly complicates the analysis of data because as the number of incorrect reads goes up, the probability of these incorrect reads also being present in multiple copies also goes up. In the case of a single copy nuclear gene in a diploid organism, the maximum number of alleles that could be present is two, which reduces the bioinformatic problem. In the case where there are an unknown number of alleles, such as for MHC I, it may not be possible to determine the real alleles even with very high coverage because the frequency of reads that represent errors may grade into the frequency of reads reflecting real alleles with poor relative amplification success.

Illumina and Ion Torrent platforms are increasing their read lengths, and are now or soon will be useful for sequencing through entire PCR products. Each platform has a different rate and pattern of errors^1^,^20^,^21^. The sequences analyzed here were generated on the Roche 454 platform. However, we expect the observed large differences in enzyme performance to be evident on the other HTS platforms as well, although the exact coverage required for high confidence in the results will likely be different. New library preparation protocols that target loci without being based on PCR, such as hybridization-based enrichment, are being developed. However, they still require PCR enrichment steps, so even with these protocols, enzyme choice is important and can affect sequencing results.

Ideally, the necessary coverage for a particular system should be calculated based on the observed bias in allele amplification and errors in the enzyme and platform combination used in a particular experiment. In planning a HTS project it is also important to keep in mind that the numbers for coverage presented here to have confidence in a particular haplotype or genotype are not average numbers for a project, but minimum coverage numbers for each sample in a study. Since the coverage of all individuals analyzed simultaneously in a run is never exactly equal, the average coverage that should be planned for in a study would thus be higher.

## Methods

### Samples

We used DNA samples from three gray wolves (*Canis lupus*) from which the 5′ end of the mitochondrial control region had been Sanger sequenced in previous studies, and thus was known^22^,^23^. The loci had different GC content, from 44% to 66%, and the longest homopolymer was 5 bp (present in at least one allele of each locus). Three Retuertas breed domestic horses (*Equus caballus*) with known MHC genotypes (Brandariz-Fontes *et al.* in preparation) were selected for the nuclear loci tests. Each DNA sample was quantified using a NanoDrop ND-1000 Spectrophotometer (NanoDrop Technologies, Inc., Wilmington, DE, USA), and the concentration was adjusted to 10 or 30 ng/μl for subsequent PCR amplifications.

### Taq polymerase

A range of 13 high fidelity, regular, economy and premium *Taq* polymerase enzymes were selected: Biotaq® (Bioline, London, UK), FastStart® High Fidelity PCR System (Roche, Mannheim, Germany), AmpliTaq Gold® (Applied Biosystems, Warrington, UK), HotStarTaq® DNA Polymerase (Qiagen, Hilden, Gernamy), Phusion® High Fidelity DNA Polymerase (Finnzymes, Espoo, Finland), *Taq* DNA Polymerase (Roche, Maylan, France), i-Max^TM^ II DNA Polymerase (iNtRON Biotechnology, Seongnam, Korea), KAPA HiFi™ (Kapa Biosystems, Boston, USA), One*Taq*™ DNA Polymerase (New England Biolabs, Hitchin, UK), Vent® DNA Polymerase (New England Biolabs, Hitchin, UK), Deep Vent® DNA Polymerase (New England Biolabs, Hitchin, UK), Pwo® DNA Polymerase (Roche, Maylan, France) and Velocity DNA Polymerase (Bioline, London, UK) (abbreviated names in Table 1). The list price of these enzymes for the amount recommended for a single 10 μl reaction (not including tax, handling or shipping) ranged from €0.01 to €0.63 (Spain, June 2013).

### Assessment of accuracy for different enzymes

Loci for Tests 1–3 were amplified in a two-step process following the universal tailed amplicon design proposed by Roche^24^,^25^. First, loci were amplified with locus-specific primers with an M13 tail, and then a Multiplex Identifier (MID) and the sequencing primer were added in a second-round PCR using the same enzyme as for the first PCR. For Test 1, the 5′ end of the wolf mitochondrial control region was amplified with the primers Thr-L^22^ and ddl5^26^, which target a 168–172 bp fragment excluding primers (variation due to indels). For Test 2, a 257 bp fragment of MHC II in horse was amplified with primers Be3 and Be4^27^. For Test 3, a 184 bp fragment of MHC I was amplified using primers PpLAa2U270 and Ppa2L542^28^. In the second PCR, we used the first PCR as a template with a 52 bp primer which included the M13, a sample-specific 10 bp MID, the 454 Sequencing System Primer sequence and a 4 bp primer key (Table 2).

All reactions were prepared in 10 μl using the standard PCR conditions following the manufacturer's protocols that came with each enzyme for both PCR steps. These were 40 cycles of: 15 or 30 seconds at 94–98°C, 20, 30 or 90 seconds at 58°C, and 30, 60 or 90 seconds at 72°C; with a final extension at 72°C for 5, 7 or 10 minutes. All cycling was performed on a DNA Engine Peltier Thermal Cycler. All reactions, including blank controls, were checked for amplification success on a 1.5% agarose gel and visualized with SYBR®Safe (Invitrogen, Paisley, UK). All successful first PCR products were diluted and used as templates for the second-round PCRs. Second PCR products were cleaned using Agencourt AMPure xp system (Beckman Coulter, Brea, CA, USA).

### Assessment of PCR protocols to reduce amplification errors

We repeated Tests 2 and 3 with modified cycling conditions in an attempt to reduce errors: Test 2b & 3b, respectively. The goal was to generate comparable data to evaluate the effect of the cycling conditions on the accuracy of the sequences (Test 2 vs 2b; Test 3 vs 3b). For the first and second PCRs the number of cycles was reduced to 25, the elongation time within cycles increased to 180 seconds and the final extension step was eliminated. Similar amplifications conditions have been suggested previously in the literature to reduce errors during amplification steps^13^,^14^,^29^,^30^,^31^,^32^. All the cycling reactions were performed on a DNA Engine Peltier Thermal Cycler. All reactions, including blank controls, were checked for amplification success on a 1.5% agarose gel and visualized with SYBR®Safe (Invitrogen). All successful first PCR products were diluted and used as templates for the second-round PCRs. Second PCR products were cleaned using Agencourt AMPure xp system (Beckman Coulter, Brea, CA, USA).

### Library preparation and sequencing

Purified PCR products from all tests were quantified using Quant-it PicoGreen dsDNA Assay Kit (Invitrogen) in a Light Cycler 480 II real-time PCR machine (Roche). Then they were adjusted to equimolar concentration (2x10^5^ molecules/μl in TE buffer) and all amplification products were pooled together. The pool was then quantified using the Quant-it PicoGreen dsDNA Assay Kit (Invitrogen) on a QuantiFluor^TM^-ST fluorometer (Promega, US).

Emulsion PCR was performed according to the manufacturer's instructions with GS Junior Titanium emPCR Kit Lib-A (Roche) and sequenced in a single 454 Roche Junior run.

### Data Analysis

Reads containing the complete target primers and barcodes were extracted from the multifasta output file and de-multiplexed on the basis of the barcode and loci specific primer sequences using jMHC^33^. The different sequences were compared to the known haplotype or genotype to determine correct sequences in Geneious v6.1.7 (Biomatters, Auckland, NZ). These previously known sequences were the reference against which the sequences identified in jMHC were compared, and reads were considered to have the correct sequence when it was identical to the reference. The proportion of correct reads was calculated by dividing the number of reads with correct sequences by the total number of reads from a particular amplicon.

### Statistical analysis

We evaluated the effect of enzymes on the proportion of correct reads with generalized linear mixed models (GLMM), using the function lmer from the *lme4* package^34^ in R (Bates, D., Maechler, M. & Bolker, B. lme4: Linear mixed-effects models using S4 classes. R package version 0.999999-0. (2012); R Core Team R: A language and environment for statistical computing. R Foundation for Statistical Computing, Vienna, Austria. http://www.R-project.org/ (2013)) for each test separately. Only cases with more than 10 reads per individual test were included in the analysis. The *Taq* polymerase was included as a fixed effect and individual as a random effect. We also used a GLMM with lmer function to evaluate the effect of the PCR protocol (standard/modified) on the proportion of correct reads for the medium and complex systems (Test 2 vs Test 2b; Test 3 vs Test 3b). PCR condition was included as a fixed effect, and enzyme and individual as random effects. We tested the significance of the variables by comparing different models using ANOVAs.

We prepared a script in Python 2.7.4 to calculate the probability of obtaining a minimum of three reads with the correct sequences for the different *Taq* enzymes when varying the total number of reads for a single haplotype and for one locus with two alleles (the script is available in Supplementary file 2, online). These probabilities are based on the frequency of correct reads observed per enzyme in Test 1 for the case of the single haplotype, and Tests 2 & 2b combined for the case of one locus with two alleles. Simulations were run only on datasets with >10 reads. We considered the number of reads when this probability reached 99.9% as an indication of the coverage needed with a given enzyme to be able to reliably identify the correct haplotype or alleles in a genotype. Often, the different alleles in multi-allelic systems do not amplify equally within a reaction. For this reason we also calculated the probability of obtaining three reads with the correct sequence for each allele when one amplifies half as well as the other.

## Author Contributions

J.A.L. conceived the experiment, C.B.-F. did the lab work under C.R. and J.A.L. supervision, M.C.-S. and C.V. did the statistics, J.A.L. wrote the manuscript with C.B.-F., M.C.-S. and C.V.A. All authors contributed preparation of the final draft, and approved it (J.A.L., C.B.-F., M.C.-S., C.V.A., C.R. and J.L.V.-P.). C.B.-F. and M.C.-S. contributed equally.

## Supplementary Material

Supplementary Information Supplementary Files

## Figures and Tables

**Table 1:** Coverage necessary to reach a 99.9% probability of recovering three copies of the correct sequence for all alleles (based on the proportion of correct reads). Since not all alleles in a PCR amplify equally well, we calculated the coverage needed when two alleles amplify at the same rate (equal amplification), and when one allele yields twice as many products as the other (unequal amplification). Enzymes that did not amplify are marked n.a. and those which amplified but for which there was insufficient data to calculate coverage are labeled i.d. Abbreviations are those used in Figures 1 and 2 and the text

Enzyme	Abbreviation	Test 1	Test 2 equal amplification	Test 2 unequal amplification
Phusion® High Fidelity DNA Polymerase (Finnzymes)	Phusion	7	42	87
KAPA HiFi™ (Kapa Biosystems)	KapHF	7	i.d.	i.d.
Pwo® DNA Polymerase (Roche)	Pwo	7	n.a.	n.a.
AmpliTaq Gold® (Applied Biosystems)	Gold	9	48	88
i-MaxTM II DNA Polymerase (iNtRON Biotechnology)	iMax	11	57	99
Taq DNA Polymerase (Roche)	Roche Taq	11	120	185
Velocity DNA Polymerase (Bioline)	Velocity	12	n.a.	n.a.
HotStarTaq® DNA Polymerase (Qiagen)	HotStar	14	97	152
FastStart® High Fidelity PCR System (Roche)	FastStart	14	45	86
Biotaq® (Bioline)	Biotaq	16	271	395
OneTaq™ DNA Polymerase (New England Biolabs)	OneTaq	i.d.	n.a.	n.a.
Vent® DNA Polymerase (New England Biolabs)	Vent	n.a.	n.a.	n.a.
Deep Vent® DNA Polymerase (New England Biolabs)	DeepVent	n.a.	n.a.	n.a.

**Table 2:** Primers used in first and second round reactions for all tests. We used published primers (references in text) upon which an M13 tail was added (shown in lower case). MIDs 1–96^25^ were used in both the forward and reverse primers

Test	Primer	Sequence 5′ – 3′
Test 1	Thr-L-t	gttttcccagtcacgacGAATTCCCCGGTCTTGTAAACC
Test 1	ddl5-t	aacagctatgaccatgCATTAATGCACGACGTACATAGG
Test 2	PpLAa2U270 -t	gttttcccagtcacgacGCTTCTCATCCTAGTTCCCTT
Test 2	Ppa2L542-t	aacagctatgaccatgGCCTAGGAGTGCAGCAGA
Test 3	Be3-t	gttttcccagtcacgacGGGTCTCACACCYKCCAG
Test 3	Be4-t	aacagctatgaccatgGMGCWGCAGSGTCTCYTT
Second round	forward	CGTATCGCCTCCCTCGCGCCATCAG[*MID*]gttttcccagtcacgac
Second round	reverse	CTATGCGCCTTGCCAGCCCGCTCAG[*MID*]aacagctatgaccatg
