# Supplementary material for: Effect of the enzyme and PCR conditions on the quality of high-throughput DNA sequencing results
Source: Sci Rep. 2015 Jan 27;5:8056. doi: 10.1038/srep08056 (PMC4306961; doi:10.1038/srep08056)
Supplement: Supplementary Information — Supplementary Files [file srep08056-s1.pdf]

Effect of the enzyme and PCR conditions on the quality of high-throughput DNA sequencing results

Claudia Brandariz-Fontes[§], Miguel Camacho-Sanchez[§], Carles Vilà, José Luis Vega-Pla, Ciro Rico, and Jennifer A. Leonard

[§]equal contribution

Supplementary File 1. Table of data used to calculate coverage necessary to obtaine three correct reads with high confidence. Rows marked in red were not used for calculations because fo too little data. Abbreviations for enzymes as in Table 1.

TEST	ENZYME	INDIVIDUAL	PCR Condition	INITIAL READS	CORRECT READS	% CORRECT READS	INITIAL DIF. ALLELES	CORRECT DIF. ALLELES	% CORRECT DIF. ALLELES
1	Biotaq	1	1	257	137	53.307393	105	1	0.952380952
1	Biotaq	2	1	355	188	52.95774648	136	1	0.735294118
1	Biotaq	3	1	270	146	54.07407407	103	1	0.970873786
1	FSHF	1	1	280	168	60	103	1	0.970873786
1	FSHF	2	1	0	0	0	0	0	0
1	FSHF	3	1	133	71	53.38345865	59	1	1.694915254
1	Gold	1	1	71	62	87.32394366	10	1	10
1	Gold	2	1	95	67	70.52631579	24	1	4.166666667
1	Gold	3	1	977	690	70.62436029	181	1	1.104972376
1	HotStQ	1	1	17554	11068	63.05115643	2137	1	0.046794572
1	HotStQ	2	1	1379	762	55.25743292	366	1	0.273224044

1	HotStQ	3	1	728	408	56.04395604	250	1	0.8
1	Phus	1	1	169	149	88.16568047	13	1	15.38461538
1	Phus	2	1	2	1	50	2	1	50
1	Phus	3	1	1	1	100	1	1	100
1	TaqRoche	1	1	292	209	71.57534247	68	1	1.470588235
1	TaqRoche	2	1	769	517	67.23016905	179	1	0.558659218
1	TaqRoche	3	1	0	0	0	0	0	0
1	ImaxII	1	1	332	239	71.98795181	78	1	1.282051282
1	ImaxII	2	1	2439	1590	65.19065191	415	1	0.240963855
1	ImaxII	3	1	236	149	63.13559322	71	1	1.408450704
1	KapHF	1	1	37	34	91.89189189	4	1	25
1	KapHF	2	1	494	399	80.76923077	58	1	1.724137931
1	KapHF	3	1	254	217	85.43307087	28	1	7.142857143
1	Pwo	1	1	1046	945	90.34416826	70	1	1.428571429
1	Pwo	2	1	257	219	85.21400778	29	1	3.448275862
1	Pwo	3	1	273	220	80.58608059	38	1	2.631578947
1	OneTaq	2	1	14	7	50	8	1	12.5
1	Velocity	2	1	389	252	64.781491	94	1	1.063829787
2	Biotaq	1	1	337	8	2.37388724	57	2	3.50877193

2	Biotaq	1	2	1732	63	3.637413395	195	2	1.025641026
2	Biotaq	2	1	278	32	11.51079137	113	1	0.884955752
2	Biotaq	2	2	9856	1702	17.26866883	2203	1	0.045392646
2	Biotaq	3	1	388	20	5.154639175	119	1	0.840336134
2	Biotaq	3	2	128	13	10.15625	52	1	1.923076923
2	FSHF	1	1	738	468	63.41463415	172	2	1.162790698
2	FSHF	1	2	312	214	68.58974359	92	2	2.173913043
2	FSHF	2	1	419	313	74.70167064	84	1	1.19047619
2	FSHF	2	2	267	163	61.04868914	94	1	1.063829787
2	FSHF	3	1	468	346	73.93162393	91	1	1.098901099
2	FSHF	3	2	38	23	60.52631579	16	1	6.25
2	Gold	1	1	141	90	63.82978723	51	2	3.921568627
2	Gold	1	2	5	4	80	3	2	66.66666667
2	Gold	2	1	185	117	63.24324324	60	1	1.666666667
2	Gold	2	2	108	69	63.88888889	39	1	2.564102564
2	Gold	3	1	455	265	58.24175824	166	1	0.602409639
2	Gold	3	2	173	111	64.16184971	59	1	1.694915254
2	HotStQ	1	1	79	32	40.50632911	47	2	4.255319149
2	HotStQ	1	2	102	23	22.54901961	78	2	2.564102564

2	HotStQ	2	1	49	18	36.73469388	28	1	3.571428571
2	HotStQ	2	2	1369	340	24.83564646	832	1	0.120192308
2	HotStQ	3	1	6	0	0	6	0	0
2	HotStQ	3	2	83	20	24.09638554	60	1	1.666666667
2	Phus	1	1	107	79	73.8317757	26	2	7.692307692
2	Phus	1	2	125	104	83.2	21	2	9.523809524
2	Phus	2	1	228	192	84.21052632	31	1	3.225806452
2	Phus	2	2	222	194	87.38738739	28	1	3.571428571
2	Phus	3	1	44	37	84.09090909	8	1	12.5
2	Phus	3	2	315	261	82.85714286	53	1	1.886792453
2	TaqRoche	1	1	118	6	5.084745763	47	2	4.255319149
2	TaqRoche	1	2	130	23	17.69230769	35	2	5.714285714
2	TaqRoche	2	1	97	21	21.64948454	60	1	1.666666667
2	TaqRoche	2	2	37	15	40.54054054	18	1	5.555555556
2	TaqRoche	3	1	304	68	22.36842105	160	1	0.625
2	TaqRoche	3	2	25	9	36	11	1	9.090909091
2	ImaxII	1	1	87	45	51.72413793	38	2	5.263157895
2	ImaxII	2	1	4	3	75	2	1	50
2	ImaxII	3	1	334	150	44.91017964	139	1	0.71942446

2	KapHF	1	1	0	0	0	0	0	0
2	KapHF	2	1	3	1	33.33333333	2	1	50
2	KapHF	3	1	1	0	0	1	0	0
3	Biotaq	1	1	152	29	19.07894737	136	16	11.76470588
3	Biotaq	1	2	339	117	34.51327434	236	29	12.28813559
3	Biotaq	2	1	253	59	23.3201581	204	18	8.823529412
3	Biotaq	2	2	1	1	100	1	1	100
3	Biotaq	3	1	202	38	18.81188119	178	21	11.79775281
3	Biotaq	3	2	81	36	44.44444444	68	23	33.82352941
3	FSHF	1	1	1352	763	56.43491124	536	30	5.597014925
3	FSHF	1	2	576	335	58.15972222	240	30	12.5
3	FSHF	2	1	703	407	57.89473684	290	27	9.310344828
3	FSHF	2	2	152	80	52.63157895	90	22	24.44444444
3	FSHF	3	1	497	297	59.75855131	221	29	13.12217195
3	FSHF	3	2	33	16	48.48484848	24	8	33.33333333
3	Gold	1	1	547	215	39.30530165	343	29	8.454810496
3	Gold	1	2	60	34	56.66666667	34	10	29.41176471
3	Gold	2	1	92	50	54.34782609	64	22	34.375
3	Gold	2	2	191	120	62.82722513	94	23	24.46808511

3	Gold	3	1	150	75	50	94	23	24.46808511
3	Gold	3	2	89	50	56.17977528	55	17	30.90909091
3	HotStQ	1	1	148	49	33.10810811	121	25	20.66115702
3	HotStQ	1	2	11	6	54.54545455	8	3	37.5
3	HotStQ	2	1	109	29	26.60550459	90	15	16.66666667
3	HotStQ	2	2	27	8	29.62962963	24	5	20.83333333
3	HotStQ	3	1	20	4	20	19	4	21.05263158
3	HotStQ	3	2	105	24	22.85714286	86	12	13.95348837
3	Phus	1	1	66	43	65.15151515	37	14	37.83783784
3	Phus	1	2	1404	859	61.18233618	446	31	6.950672646
3	Phus	2	1	157	95	60.50955414	71	14	19.71830986
3	Phus	2	2	2038	1344	65.94700687	614	30	4.885993485
3	Phus	3	1	44	21	47.72727273	35	12	34.28571429
3	Phus	3	2	652	407	62.42331288	226	30	13.27433628
3	TaqRoche	1	1	35	6	17.14285714	28	4	14.28571429
3	TaqRoche	1	2	129	43	33.33333333	97	16	16.49484536
3	TaqRoche	2	1	122	26	21.31147541	102	13	12.74509804
3	TaqRoche	2	2	115	53	46.08695652	80	18	22.5
3	TaqRoche	3	1	298	77	25.83892617	229	24	10.48034934

3	TaqRoche	3	2	66	26	39.39393939	48	11	22.91666667
3	ImaxII	1	1	19	12	63.15789474	15	8	53.33333333
3	ImaxII	2	1	183	94	51.36612022	112	26	23.21428571
3	ImaxII	3	1	2091	1098	52.5107604	859	30	3.492433062
3	KapHF	1	1	864	613	70.94907407	241	31	12.86307054
3	KapHF	2	1	464	332	71.55172414	146	28	19.17808219
3	KapHF	3	1	305	216	70.81967213	111	30	27.02702703
1: mitDNA		1:Ret17 or CVA390	1:Q1/Estándar PCR Condition	INITIAL TOTAL NUMBER OF READS	CORRECT NUMBER OF READS	% CORRECT READS	INITIAL NUMBER OF DIFERENT ALLELES	CORRECT NUMBER OF DIFERENT ALLELES	% CORRECT NUMBER OF ALLELES
2:MHC classII (DRA)		2:Ret280 or JAL4914	2:Q2/Reduced PCR						
3: MHC class I (b3)		3:Ret98 or JAL4934b							

Effect of the enzyme and PCR conditions on the quality of high-throughput DNA sequencing results

Claudia Brandariz-Fontes[§], Miguel Camacho-Sanchez[§], Carles Vilà, José Luis Vega-Pla, Ciro Rico, and Jennifer A. Leonard

Supplementary File 2: Scripts

SCRIPTS

Python 2.7.4 scripts and empirical data used in the manuscript "*Effect of the enzyme and PCR conditions on the quality of high-throughput DNA sequencing results*" by Brandariz-Fontes, Camacho-Sanchez, Vilà, Vega-Pla, Rico and Leonard. These scripts were prepared to run in a computer under the operative system Ubuntu 13.4.

`mtDNA.py`: Python script to calculate the probability of obtaining at least a given number (for example 3, variable provided by the user) of copies of the correct mtDNA haplotype when analyzing a variable number of next generation sequencing reads obtained after a PCR amplification using different enzymes. The output is presented in a file with the suffix "`_out`". The number of reads needed for each enzyme to reach a user-defined probability (for example 99.9%) of amplifying that number of copies is provided in a separate file with the suffix "`_Thresholds`".

`2alleles_genotypes.py`: As above, but for a system with 2 alleles: probability of obtaining a given number of copies of the two alleles. Since the amplification success often differs among alleles, the user can define a lower amplification success for one allele.

`mtDNA_enzyme_data` and **`2alleles_enzyme_data`**: Empirical estimate of the proportion of reads with the correct sequence for mtDNA and for a system with 2 alleles, respectively, used as input for the previous scripts. The files contain two columns separated by one Tab. The first column includes the name of the enzyme and the second, the proportion of reads with the correct sequences.

`mtDNAtest.py`

```
#!/usr/bin/env python

# Program to calculate the probability of obtaining at least three (a variable number,
# variable name: "Good") correct sequences out of a sample of a variable number ("k")
# of reads obtained from next generation sequencing projects.
# Input will the the probability of one sequencing being correct ("Pgood"), as estimated
# from empirical data.
# The program registers at which number of reads the probability reaches a certain
```

```
# threshold ("Threshold"), for example 99.9%

from math import factorial                            # Import package to calculate factorials



# Input 1: a text file containing in each line the name of an enzyme and the proportion
# of correct sequences obtained with it.
DataFileName= raw_input("Name of file with enzyme info: ")

# Input2: Minimum number of identical sequences to trust that this is the correct
# sequence. It is assumed to always be the most frequent sequence.
Good= int(raw_input("Minimum number of identical reads required to trust that this is a true allele: "))

# Input3: Threshold probability (percent) that we require to be sure that we obtain
# that number of correct reads
Threshold= float(raw_input("Threshold probability (%) of obtaining that minimum number of correct reads (ej. 99.9): "))/100

# Input4: Maximum number of reads that we want to consider to reach "Good"
Max= int(raw_input("Maximum number of reads to consider: "))

# Open file with the enzyme info (2 columns, separated by a tab)
InFile= open(DataFileName, 'r')



# Open file for output. The file will have the same name as the input file but with
# the suffix "_Thresholds"
OutFileName=DataFileName+"_Thresholds"
Output=open(OutFileName,'w')

# Write information used for this run in the output file
print >> Output, "Data File: ", DataFileName
print >> Output, "Minimum number of identical reads required to trust an allele: ", Good
print >> Output, "Target minimum probability (%) of getting at least this number of correct reads: ", Threshold*100, "%"
print >> Output, "Maximum number of reads considered: ", Max, "\n"
```

```
# Create an array where the probabilities will be added.
EnzymeList= []

EnzymeCounter=0
for Line in InFile:
      # Read, line by line, the file with the enzyme info

      EnzymeCounter=EnzymeCounter+1
      # Remove line ending characters
      Line = Line.strip('\n')
      # Split the line into a list of elements, using tab as a delimiter
      ElementList = Line.split('\t')

      # Identify enzyme name and probability
      Enzyme = ElementList[0]
      Pgood = float(ElementList[1])

      # Create an array where the probabilities will be added.
      EnzymeList.append(Enzyme)

      # Initialize value for the number of reads needed to reach "Threshold"
      kneeded=0

      # Increase the number of reads from "Good" to the maximum number of sequences to consider ("Max")
      for k in range(Good,Max+1):

            # Calculate the probability of obtaining less than "Good" (for example 3) good sequences
            P=0
            for l in range(0,Good):       # Probability of obtaining a number of correct sequences under the desired value
                  P=P+(factorial(k)/(factorial(l)*factorial(k-l)))*((Pgood**l)*(1-Pgood)**(k-l))

            # Probability of getting the desired number or more
            P=1-P

            # Export the value into the array with probabilities
```

```
            EnzymeList.append(P)

            # Check if the probability is over the "Threshold". If this probability is the first
            # one in the series reaching this value, keep "k" as the number of reads needed
            if (kneeded==0) and (P >= Threshold):
                  kneeded= k

      # Print the result for each enzyme in the output file
      print >> Output, "Enzyme: ",Enzyme,", Number of reads needed: ",kneeded

InFile.close()
Output.close()





# Open file for output probabilities for each enzyme and increasing number of reads
# This file will have the same name as the input file but with the suffix "_out"
OutFileName=DataFileName+"_out"
Output2=open(OutFileName,'w')

# Convert file into a format suitable for spreadsheets (for example, to plot curves)
# Columns separated by one tab
for i in range(0,Max-Good+2):
      if i==0:
            print >> Output2, " ",
      else:
            print >> Output2, i+Good-1,         # First column in first line, empty
                                                            # In the following lines, number of reads sampled

      for j in range(0,EnzymeCounter):
            print >> Output2, "\t",EnzymeList[i+j*(Max-Good+2)],
                                                            # First line will have enzyme names, the other lines
                                                            # the corresponding probabilities for the number
                                                            # of reads
      print >> Output2,""
```

```
Output2.close()

# Indicator that the program has completed the run
print "Done!"
```

2alleles_genotypes.py

```
#!/usr/bin/env python

# Program to calculate the probability of obtaining at least three (a variable number, defined
# by the variable "Good") correct sequences for each of two alleles (diploid organism) out of
# a sample of a variable number("k") of reads obtained from next generation sequencing projects.
# Input will the probability of one sequencing being correct -one of the two valid alleles -
# ("Pgood"), as estimated from empirical data.
# The program also registers at which number of reads the probability reaches a certain threshold
# ("Threshold"), for example 99.9%

# In order to correct for differences in the amplification success among the two alleles in
# biparentally inherited loci, this program calculates the probability of obtaining that number of
# correct sequences for the least easily amplified of the two alleles.
# We assume that this allele is obtained with an amplification success that is 1 if it is
# the same as the most common allele, 0.8 if it is amplified 20% less. This is indicated by a
# proportion ("Rate2ndAllele") which could be obtained from the empirical data.

from math import factorial                           # Import package to calculate factorials



# Input 1: a text file containing in each line the name of an enzyme and the proportion
```

```
# of correct sequences obtained with it.
DataFileName= raw_input("Name of file with enzyme info: ")

# Input 2: Minimum number of identical sequences to trust that this is the correct sequence.
# It is assumed to always be the most frequent sequence that appears in the reads.
Good= int(raw_input("Minimum number of identical reads required to trust that this is a true allele: "))

# Input 3: Threshold probability (percent) that we want to be sure that we obtain
# that number of correct reads
Threshold= float(raw_input("Threshold probability (%) of obtaining that minimum number of correct reads (ej. 99.9): "))/100

# Input 4: Maximum number of reads that we want to consider to reach "Good"
Max= int(raw_input("Maximum number of reads to consider: "))

# Input 5: Rate of amplification of the second (least frequent) allele compared to the first
Rate2ndAllele= float(raw_input("Amplification success of the 2nd allele compared to the 1st (0 to 1; 1: equal amplification): "))

# Proportion of the correct alleles that correspond to the second (least frequent) allele
P2ndAllele=Rate2ndAllele/(1+Rate2ndAllele)



# Open file with the enzyme info (2 columns, separated by a tab)
InFile= open(DataFileName, 'r')

# Open file for output. The file will have the same name as the input file but with
# the suffix "_Thresholds"
OutFileName=DataFileName+"_Thresholds"
Output=open(OutFileName,'w')

# Write information used for this run in the output file
print >> Output, "Data File: ", DataFileName
print >> Output, "Minimum number of identical reads required to trust an allele: ", Good
print >> Output, "Target minimum probability (%) of getting at least this number of correct reads: ", Threshold*100, "%"
print >> Output, "Maximum number of reads considered: ", Max
print >> Output, "Relative amplification of the 2nd allele compared to the 1st: ", Rate2ndAllele
print >> Output, "         -> Proportion of the correct reads that correspond to the 2nd allele: ", P2ndAllele, "\n"
```

```
# Create an array where the probabilities will be added
EnzymeList= []

EnzymeCounter=0
for Line in InFile:
      # Read, line by line, the file with the enzyme info

      EnzymeCounter=EnzymeCounter+1
      # Remove the line ending characters
      Line = Line.strip('\n')
      # Split the line into a list of elements, using tab as a delimiter
      ElementList = Line.split('\t')

      # Identify enzyme name and probability
      Enzyme = ElementList[0]
      Pgood = float(ElementList[1])

      # Proportion of the total number of reads corresponding to each allele
      Prop2nd=Pgood*P2ndAllele           # for the second (rare) allele
      Prop1st=Pgood*(1-P2ndAllele)  # for the first (common) allele

      # Create an array where the probabilities will be added
      EnzymeList.append(Enzyme)


      # Initialize value for the number of reads needed to reach "Threshold"
      kneeded=0

      # Increase the number of reads from 2*"Good" to the maximum number of sequences to consider ("Max")
      for k in range(2*Good,Max+1):

            # Probability of obtaining at least "Good" copies of the correct alleles
            P=0
            Ptot=0
```

```
        for kgood in range(Good,k-Good+1):
            # Probability of "kgood" copies of the first allele (the one with higher amplification success)
            P1st=(factorial(k)/(factorial(kgood)*factorial(k-kgood)))*((Prop1st**kgood)*(1-Prop1st)**(k-kgood))

            # Probability of obtaining less than "Good" (for example 3) good sequences
            # for the second (rare) allele
            P2nd=0
            for l in range(0,Good):      # Probability of obtaining a number of correct sequences under the desired value
                P2nd=P2nd+(factorial(k-kgood)/(factorial(l)*factorial(k-kgood-l)))*((Prop2nd**l)*(1-Prop2nd)**(k-kgood-l))
            # Probability of getting the desired number or more
            P2nd=1-P2nd

            # Probability of getting "kgood" copies of the first allele and "Good" or more
            # of the second:
            Ptot=P1st*P2nd

            # For all possible values of the number of alleles
            P=P+Ptot

        # Export the value into the array with probabilities
        EnzymeList.append(P)

        # Check if the probability is over the "Threshold". If this probability is the first
        # one in the series reaching this value, keep "k" as the number of reads needed
        if (kneeded==0) and (P >= Threshold):
            kneeded= k

    # Print the result for each enzyme in the output file
    print >> Output, "Enzyme: ",Enzyme,
    if kneeded==0:
        print >> Output, ", Not enough with ",Max," reads"
    else:
        print >> Output, ", Number of reads needed: ",kneeded


# Open file for output probabilities for each enzyme and increasing number of reads
# This file will have the same name as the input file but with the suffix "_out"
```

```
OutFileName=DataFileName+"_out"
Output2=open(OutFileName,'w')

# Convert file into a format suitable for spreadsheets (for example, to plot curves)
# Columns separated by one tab
for i in range(0,Max-2*Good+2):
      if i==0:
            print >> Output2, " ",
      else:
            print >> Output2, i+2*Good-1,       # First column in first line, empty
                                                            # In the following lines, number of reads sampled

      for j in range(0,EnzymeCounter):
            print >> Output2, "\t",EnzymeList[i+j*(Max-2*Good+2)],
                                                            # First line will have enzyme names, the other lines
                                                            # the corresponding probabilities for the number
                                                            # of reads
      print >> Output2,""

Output2.close()


# Indicator that the program has completed the run
print "Done!"
```

mtDNA_enzyme_data

```
Biotaq      0.5344640452
FastStart   0.5669172932
Gold        0.7615820658
HotStar     0.5811751513
Phusion     0.8816568047
Roche_Taq   0.6940275576
iMax        0.6677139898
KapHF       0.8603139784
Pwo         0.8538141888
Velocity    0.6478149100
```

2alleles_enzyme_data

```
Biotaq      0.0897922490
FastStart   0.6908834575
Gold        0.6257913393
HotStar     0.2603515899
Phusion     0.8255514822
Roche_Taq   0.2065449178
iMAX        0.4831715879
```
